# Mechanical Properties of Small Clear Specimens of *Eucalyptus globulus* Labill

**DOI:** 10.3390/ma13040906

**Published:** 2020-02-18

**Authors:** Jorge Crespo, Almudena Majano-Majano, Antonio José Lara-Bocanegra, Manuel Guaita

**Affiliations:** 1 Department of Agroforestry Engineering, University of Santiago de Compostela, c/ Benigno Ledo, 27002 Lugo, Spain; jorge.crespo@usc.es (J.C.); m.guaita@usc.es (M.G.); 2 Department of Building Structures and Physics, ETS Architecture, Universidad Politécnica de Madrid, Avda Juan de Herrera 4, 28040 Madrid, Spain; antoniojose.lara@upm.es

**Keywords:** *Eucalyptus globulus*, mechanical properties, small clear specimen, bending, tension, compression, shear, friction, wood

## Abstract

*Eucalyptus globulus* Labill stands out as one of the hardwood species produced in Europe with prominent mechanical properties, which is undergoing a growing interest in extending added value. The development of engineered wood products with this species and its application in timber structures involving numerical finite element simulations requires knowledge of the mechanical properties for the different orthotropic material directions. The aim of the present study is to determine the main mechanical properties of *E. globulus* from small clear specimens, necessary for the development of finite element models. The work provides experimental results on the ultimate capacity and modulus of elasticity considering different stresses: tension parallel and perpendicular to the grain, compression parallel and perpendicular to the grain (in radial and tangential directions), shear and longitudinal static bending. The work is complemented with experimental data on timber-to-timber friction coefficients for 0°, 45°, and 90° orientation angles, which are useful in the modeling of traditional joints. Very high values of ultimate stress and modulus of elasticity for the different mechanical properties were obtained, highlighting the great potential of this species for structural applications.

## 1. Introduction

The increasing political and social demands regarding the use of eco-friendly building materials are leading in recent years to a strong rise in the use of wood due to its CO_2_ absorption capacity and the quality of renewable natural resource.

There is growing interest in Europe towards glued laminated structural products made of hardwoods due to several reasons, such as the shortage of softwoods, large stocks of hardwoods, and policies of re-afforestation for several hardwood species due to better adaptation to soil and climate conditions. In most cases, these products reach greater bending strengths than those of the highest European softwood glulam strength classes [1], usually made of spruce or pine.

*Eucalyptus globulus* Labill is a temperate-climate hardwood with growth in Europe, Asia, Africa, Oceania, and America [2]. In Europe, it can be found mainly in Spain, Portugal, and Italy. Spanish *E. globulus* L. is structurally characterized [3] and can be assigned to D40 strength class, which is currently the highest strength class for European hardwood species [4]. In addition, *E. globulus* from Galicia region (Spain) shows a high natural durability against fungi, similar to that of chestnut and oak [5]. The abundance of the species and its great performance have recently lead to important experimental research using structural-size specimens with the aim of developing new structural products [6,7,8,9,10].

Numerical simulations may also be of interest in many research fields and engineering applications. For the development of finite element models (FEM) that adequately simulate the wood behavior at the macroscopic level, it is essential to know the mechanical properties [11], usually obtained from experimental tests on small clear specimens.

Although wood is an anisotropic material, the assimilation to an orthotropic one defined by three main directions, longitudinal (*L*), radial (*R*) and tangential (*T*), is generally accepted. For situations with low deformation levels far from the ultimate load, it results appropriate to assume an elastic and linear material model in the three directions [12]. However, the elastic and linear model is not suitable to estimate the ultimate load of structural elements because yielding and/or brittle failures can occur.

At present, there are many FEM softwares which offer different material models roughly adequate to simulate the elastoplastic and orthotropic behavior of wood. In any case, a preliminary required task consists in knowing the actual material behavior until failure for the different basic stresses (tension, compression, and shear) in the relevant orthotropy directions to derive the input parameters needed in any timber engineering FEM (e.g., [13,14]). Additionally, information on the friction coefficients may also be of interest, especially in the modeling of traditional timber joints [15].

Previous research by the authors in small clear specimens of *E. globulus* L. reported the twelve elastic constants of orthotropy obtained from compression testing (longitudinal elastic moduli, shear moduli, and Poisson coefficients) [16] and fracture parameters [17,18,19]. However, to our knowledge there are no studies on this species reporting complementary information necessary for the development of numerical finite element models, such as strength to different stresses and directions or friction coefficients.

This work presents the experimental results of fundamental mechanical properties of *E. globulus* L. Standardized destructive tests of tension parallel and perpendicular to the grain, compression parallel and perpendicular to the grain (in radial and tangential directions), shear and longitudinal static bending, combined with conventional strain measurement devices and a digital image correlation technique were performed using small clear specimens. Likewise, tests to determine the timber-to-timber friction coefficient for different grain orientation angles (0°, 45°, and 90°) were carried out.

## 2. Materials and Methods

### 2.1. Specimens

Planed boards of *Eucalyptus globulus* Labill from the Galicia region, northwest of Spain, cut from heartwood (with no juvenile wood), with 3000 mm × 80 mm × 20 mm dimensions and radial annual ring orientation were used for specimen preparation. The boards were conditioned at 20 °C and 65% relative humidity. Each group of tests was formed by specimens coming from different boards to take into account the variability of the material. All of the specimens were free from knots.

The moisture content (MC) of the specimens was measured by the oven-dry method according to ISO 13061-1:2014 [20], ranging between 9.0% and 11.8% with a mean value of 10.2%. The density (*ρ*) of the specimens was also determined according to ISO 13061-2:2014 [21]. The results varied between 638 and 1125 kg/m^3^, with a mean density value of 847 kg/m^3^.

### 2.2. Experimental Methods

**Figure 1:**
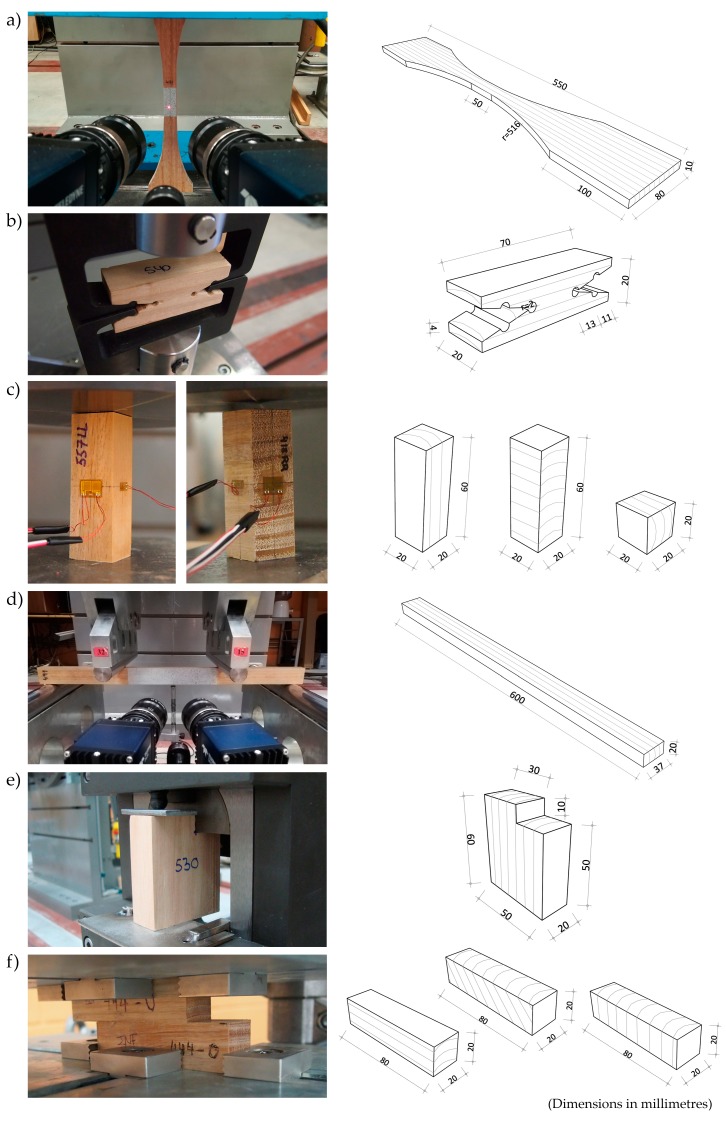
Test setup (left column) and specimen geometry (right column): (**a**) tension parallel to grain; (**b**) tension perpendicular to grain; (**c**) compression parallel and perpendicular to grain; (**d**) static bending; (**e**) shear; (**f**) timber-to-timber static friction.

The tensile stress parallel to the grain was derived from 20 tension tests executed in compliance with ISO 13061-6:2014 [22]. The specimens were curved shaped, as shown in Figure 1a, to ensure that failure occurred within the central portion and to minimize stress concentration in the transition area.

The specimens were clamped on either end, and the clear distance between the grips was at least 350 mm. The cross-sectional dimensions at the middle were 20 mm in radial direction and 10 mm in tangential direction.

In order to determine the modulus of elasticity in tension parallel to the grain, *E_t,_*_0_, strains at a central area of the specimen of 50 mm length were measured during testing using a digital image correlation (DIC) technique, ARAMIS 3D of 12 MPixels (GOM mbH, Braunschweig, Germany) [23] (Figure 1a). This is a non-contact and material-independent measuring system which provides full-field strain measurements, which is advantageous compared with other traditional techniques such as strain gauges.

Tension perpendicular to grain tests were performed in accordance with UNE 56538:1978 [24]. A total of 36 specimens were cut to the shape shown in Figure 1b with a minimum cross-section of 20 × 20 mm^2^ and oriented in such a way that loading was produced in radial direction. The ends of the specimens were clamped with symmetrical grips. The tests were executed at a constant velocity of 400 kg/min.

Compression parallel and perpendicular to the grain tests (Figure 1c) were carried out according to ISO 13061-17:2017 [25] and ISO 13061-5:2020 [26], respectively. For the cases of compression parallel to the grain, 20 prismatic specimens of 60 × 20 × 20 mm^3^ were used, and 20 of the same dimensions were used for compression perpendicular to the grain with load applied in radial direction. In the case of compression perpendicular to the grain with load applied in tangential direction, 20 specimens of 20 × 20 × 20 mm^3^ were used instead due to thickness limitation of the eucalyptus boards.

Strain gauges were located on the specimens’ faces to measure strains and derive the modulus of elasticity for each direction (see Figure 1c). Further details on the compression setup can be found in a previous work by the authors [16] which had the objective of determining the orthotropic elastic constants of eucalyptus.

The ultimate value of the stresses in axial direction (ultimate tensile stress parallel to grain, *σ_t_*_,0_; ultimate tensile stress perpendicular to grain, *σ_t_*_,90_; and ultimate stress in compression parallel to grain, *σ_c_*_,0_) were calculated from the maximum load in each batch of tests, *F*_max_, and the cross-sectional dimension at the middle of the specimen, *S*, according to Equation (1):

(1) $$\sigma_{i,\alpha }=\frac{F_{\max}}{S}$$

Regarding the compression perpendicular to grain, it is not possible to clearly determine a *F*_max_ because the load is continuously increasing in this type of test. Therefore, the proportional limit stress, *σ_c_*_,90,*y*_, and the stress at a specific deformation level (2 mm), *σ_c_*_,90,2_, were reported. *σ_c_*_,90,*y*_ was calculated as the relationship between the load at the proportional limit and the specimen cross-section. *σ_c_*_,90,2_ was determined from the load at 2 mm deformation and the specimen cross-section. The 2 mm deformation was measured from the intersection of the straight line tangent to the elastic slope of the load-deformation curve and the horizontal axis.

Static bending tests were performed following a 4-point loading configuration as specified in EN 408:2011 [27] in order to have constant moment at the central beam area (Figure 1d). The cross-section of the specimens was 20 × 37 mm^2^. The span corresponded to 15 times the specimen depth. Loads were applied at a distance of 4.5 times the depth measured from every support. A total of 22 specimens were tested.

Deflections were measured by DIC technique (ARAMIS 3D). The modulus of elasticity in bending, *E_m_*, was calculated using the relationship between the applied force and measurements of mid-span deflection relative to the supports of the test piece in accordance with Equation (2):

(2) $$E_{m}=\frac{3al^{2}-4a^{3}}{4bh^{3}\frac{w_{2}-w_{1}}{F_{2}-F_{1}}}$$

where *a* is the distance between the load application point and the nearest support; *l* is the span; *b* and *h* are the width and height of the specimen, respectively; *F*_1_ − *F*_2_ indicates the load increase on the regression line comprised in the range between 0.1*F*_max_ and 0.4*F*_max_ which provides a minimum correlation coefficient of 0.99; and *w*_2_ − *w*_1_ is the increase of deflection corresponding to *F*_1_ − *F*_2_.

The ultimate static bending stress, *σ_m_*, was calculated from the maximum load *F*_max_ following Equation (3):

(3) $$\sigma_{m}=\frac{3F_{\max}a}{bh^{2}}$$

where *b* and *h* are the width and height of the specimen, respectively, and *a* the distance between the load application point and the nearest support.

Relationships between stress and modulus of elasticity were determined for all the tests where strains could be measured. Linear trendline based on least squares methods and the corresponding *R*^2^ value were obtained for these relationships.

Shear tests were performed following UNE 56543:1988 [28]. A total of 44 specimens were prepared to the shape and dimensions indicated in Figure 1e. The loading plate was placed on the specimen notch. Part of the underside of the specimen rested on the table being 3 mm separated from the shear failure plane. The test displacement control was kept at 0.6 mm/min. The ultimate shear stress was calculated from the maximum load, *F*_max_, and the shear cross-section, *S_v_*, according to Equation (4):

(4) $$\sigma_{v}=\frac{F_{\max}}{S_{v}}$$

The static friction coefficients were determined from pairs of eucalyptus pieces oriented at 0°, 45°, and 90° with respect to the friction plane (Figure 1f) according to the procedure detailed in [29].

**Figure 2:**
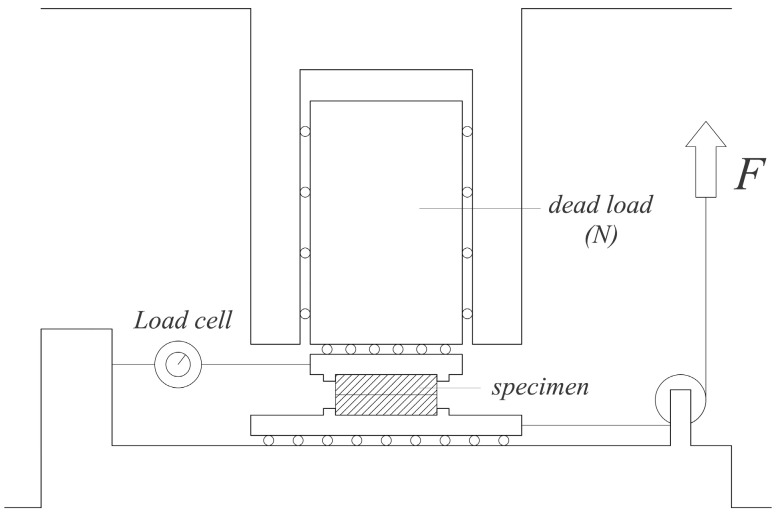
Friction tests scheme.

The dimensions of each of the two prismatic pieces that formed every specimen were 80 × 20 × 20 mm^3^. During testing, a constant vertical dead load (N) of 2.435 kN was acting on the specimen while another horizontal force (equivalent to the friction force, *F*_R_) was applied to the bottom piece (Figure 2).

The static friction coefficient between the eucalyptus pieces, *μ_s_*_,*α*_, was calculated as the relationship between the two mentioned forces (Equation (5)). A total of 130 specimens were tested.

(5) $$\mu_{s,\alpha }=\frac{F_{R}}{N}$$

## 3. Results and Discussion

**Figure 3:**
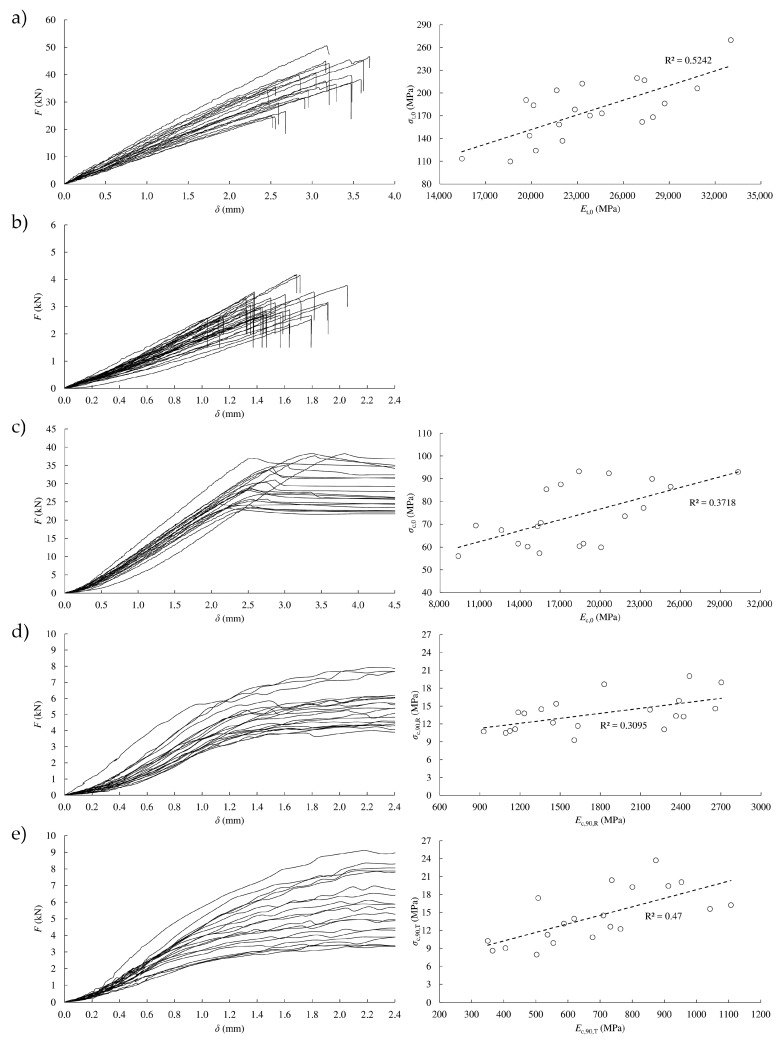
Load-displacement curves (left column) and correlation between ultimate stress and modulus of elasticity (right column): (**a**) tension parallel to grain; (**b**) tension perpendicular to grain; (**c**) compression parallel to grain; (**d**) compression perpendicular to grain in radial direction; (**e**) compression perpendicular to grain in tangential direction.

Load-displacement curves in tension parallel to the grain are shown in Figure 3a left. As can be seen, timber behaved linearly until failure in all of the tests, giving a mean ultimate stress value, *σ_t_*_,0_, of 176.3 MPa. A mean modulus of elasticity, *E_t_*_,0_, of 23.80 GPa was also attained. Both values are very high in comparison with those obtained from other softwoods. In particular, *σ_t_*_,0_ and *E_t_*_,0_ resulted to be around 2 and 2.5 times higher than the respective values attained for *Scots pine* in [30], which highlights the great mechanical performance of *Eucalyptus globulus* L.

A relatively good correlation coefficient (*R*^2^ = 0.52) was found between ultimate stress and modulus of elasticity from tension tests parallel to grain (Figure 3a right). Therefore, the modulus of elasticity could be an adequate physical property to estimate the ultimate tension stress in this direction.

**Figure 4:**
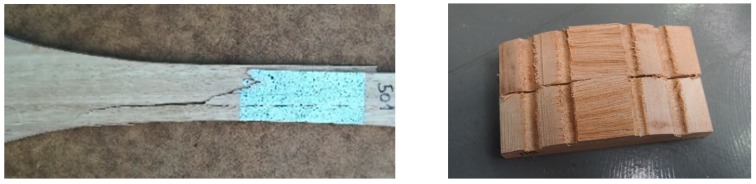
Typical failures in tension parallel to grain (**left**) and tension perpendicular to grain (**right**).

The main failure mode was identified as cross-grain tension failure in the center part of the specimen, with visible cracks extending along the direction of the grain (Figure 4 left).

In the case of tension perpendicular to grain tests, also a linear behavior until failure was exhibited (see Figure 3b). As it is known, tension perpendicular to the grain is the most critical stress in timber. Particularly, the mean value of ultimate stress resulted in 7.5 MPa, which is 23 times lower than the ultimate stress obtained in tension parallel to grain. All of the specimens showed a clean failure at the central cross-section as evinced in Figure 4 (right).

Figure 3c (left) shows the load-displacement curves in compression parallel to the grain. These curves exhibited an initial elastic domain practically up to *F*_max_ and a plastic behavior without hardening afterwards with a noticeably horizontal tangent. The mean value of the ultimate stress in compression parallel to grain, *σ_c_*_,0_, resulted in 73.6 MPa, that is, 42% of *σ_t_*_,0_. This value was much higher (in between 52% and 84%) than that obtained from analogous compression tests carried out on the four Spanish softwoods that are structurally classified (*Pinus radiata*, *Pinus pinaster*, *Pinus sylvestris,* and *Pinus nigra*) [31]. A mean value of 18.06 GPa resulted for the modulus of elasticity, *E_c_*_,0_, meaning 76% of *E_t_*_,0_. For this stress, a not very high correlation between ultimate stress and modulus of elasticity was obtained (*R*^2^ = 0.37, see Figure 3c right).

**Figure 5:**
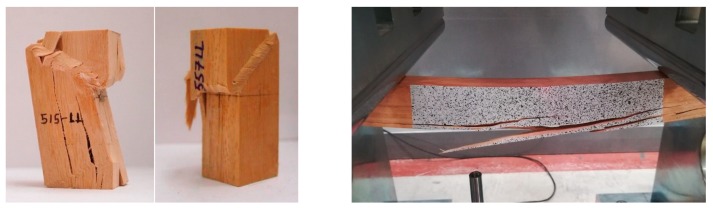
Typical failure modes in compression parallel to the grain (**left**); bending failure due to small grain deviation (**right**).

The typical failure mode exhibited by the specimens subjected to compression parallel to grain can be seen in Figure 5 (left). It is a crushing failure, typical of small specimens subjected to this type of stress. It is usually characterized by folding of the cellulose microfibrils that can begin at low stress levels [32]. Folding takes place also at the cell wall at high stress levels, and eventually leads to gross failure of the specimen.

The load-displacement curves for compression perpendicular to the grain in radial and tangential direction are depicted in Figure 3d,e, left, respectively. In these cases, the curves did not show a decrease in loading during testing, so a clearly defined maximum load cannot usually be obtained. The stress at the proportional limit and stress at 2 mm deformation were reported (see Table 1).

The mean values of proportional limit stress in compression perpendicular to the grain resulted similar for both directions (11.1 and 10.2 MPa), being approximately seven times smaller than the ultimate compression stress in parallel direction. However, notably different stiffness values were found for radial and tangential orientations (1.78 and 0.69 GPa, respectively).

In general, correlations between the respective modulus of elasticity and ultimate stress in compression resulted lower than correlations derived from tension tests (Figure 3d,e, right).

**Figure 6:**
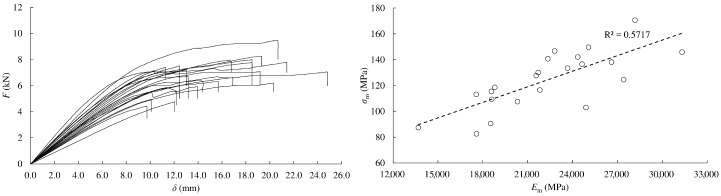
Static bending: load-displacement curves (**left**); correlation between ultimate stress and modulus of elasticity (**right**).

Load-deflection curves in static bending are shown in Figure 6 left. During testing of small clear beams, initial yielding usually occurs on the compression side, followed by compression failures. The ultimate stress is reached when brittle failure at the tensile side occurs. This is the typical bending behavior produced in small clear beams subjected to bending. The compression zone enlarges and the neutral surface shifts toward the tensile side of the beam as the tensile stress continues to increase [32]. This fact explains that mean ultimate stress and modulus of elasticity values in bending (124 MPa and 22.3 GPa, respectively) are in between tension and compression results. Even so, some of the beams presented brittle failure due to small grain deviations (cross-grain tension failure) without yielding at the compression side (Figure 5, right).

The mean ultimate bending stress of *E. globulus* was around 1.7 times higher than that obtained also from 4-point bending tests on small clear specimens of *Eucalyptus grandis x E. Urophylla* hybrids in [33]. Similarly to tension parallel to grain, a relatively good correlation coefficient (*R*^2^ = 0.57) between ultimate stress and modulus of elasticity was found in bending (Figure 6 right).

**Figure 7:**
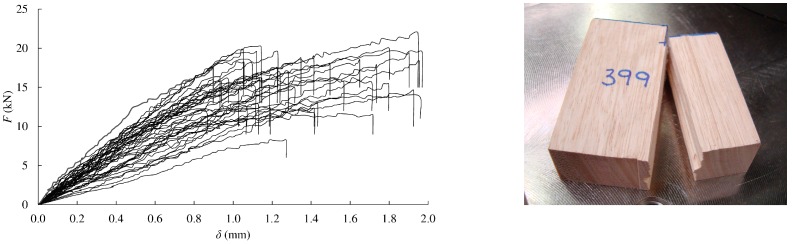
Shear: load-displacement curves (**left**); typical failure mode (**right**).

The load-displacement curves obtained from shear tests are represented in Figure 7 (left). The ultimate stress values ranged from 8.5 to 22.5 MPa, with 16.2 MPa being the mean value, around 9% of the ultimate tension stress parallel to grain. This value resulted between 1.5 and 2 times higher than those reported in [31] for the four Spanish pines following the same standard. It was also higher than most of the respective data obtained from shear block tests in a broad number of wood species compiled in [34]. A clear fracture by the shear plane was produced in all the specimens (Figure 7 right).

**Figure 8:**
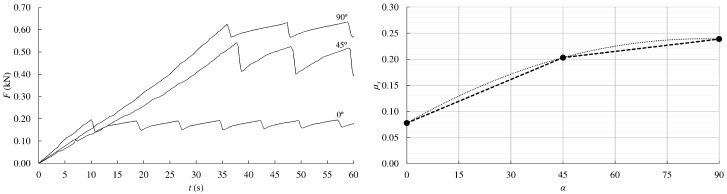
Timber-to-timber friction coefficient vs. time for 0°, 45°, and 90° grain orientations (**left**); variation of mean friction coefficients with respect to grain orientations (**right**).

Figure 8 (left) depicts the typical relationship curves between friction load and time obtained from pairs of eucalyptus pieces oriented at 0°, 45°, and 90° with respect to the friction plane. In Figure 8 (right), the mean values of static friction coefficient for the three analyzed angles are plotted. The mean friction coefficient between the eucalyptus pieces with the grain oriented parallel to the friction plane, *µ_s_*_,0_, resulted in 0.08. This value increased 2.5 times (*µ_s_*_,45_ = 0.20) when the grain formed 45° with that plane. However, the friction coefficient hardly increased for the specimens with the grain oriented at 90° in comparison with 45°, resulting in an average value of *µ_s_*_,90_ = 0.24.

The variation of friction coefficients with respect to grain direction follows a nonlinear trend. In Figure 8 (right), two curves proposals (a bilinear and a quadratic approximation) are presented to estimate the friction coefficient for different orientation angles to those studied here. Of course, more tests would be necessary to fit these curves with greater accuracy. The *µ_s_*_,90_ coefficient determined for eucalyptus resulted to be around half of the value obtained for *Picea abies* (L) Karst following the same test procedure in previous work by the authors [29].

The experimental results of all the mechanical properties are summarized in Table 1. Mean value, standard deviation (SD), and coefficient of variation (CoV) for each property are listed together with the number of tested specimens (*n*).

## 4. Conclusions

Mechanical tests on small clear specimens of *Eucalyptus globulus* L. were performed. Very high values of ultimate stresses and modulus of elasticity were obtained in comparison with common softwoods, highlighting the great potential of eucalyptus for structural applications.

The best correlations between ultimate stress and modulus of elasticity were found in bending and tension parallel to the grain. Compression tests showed the lowest correlations between both properties.

The timber-to-timber friction coefficients resulted lower than those of other species. The values followed a nonlinear trend considering different grain orientations with respect to the friction plane.

The mean values of stiffness, ultimate stresses, and friction coefficients attained are of great interest to be used as input parameters in the development of local finite element models.

## Figures and Tables

**Table 1:** Mechanical properties of *Eucalyptus globulus* L.

Mechanical Property	Symbol	*n*	Mean	SD	CoV
***Tension parallel to grain***					
Ultimate stress (MPa)	*σ_t_*_,0_	20	176.3	39.6	22%
Modulus of elasticity (GPa)	*E_t_*_,0_	20	23.80	4466	19%
***Tension perpendicular to grain***					
Ultimate stress (MPa)	*σ_t_*_,90_	36	7.5	1.2	16%
***Compression parallel to grain***					
Ultimate stress (MPa)	*σ_c_*_,0_	20	73.6	13.4	18%
Modulus of elasticity (GPa)	*E_c_*_,0_	20	18.06	5155	29%
***Compression perpendicular to grain***					
Proportional limit stress—radial (MPa)	*σ_c_*_,90,*R*,*y*_	20	11.1	2.5	22%
Modulus of elasticity—radial (GPa)	*E_c_*_,90,*R*_	20	1.78	595	33%
Stress at 2mm deformation—radial (MPa)	*σ_c_*_,90,*R*,2_	20	13.0	2.8	22%
Proportional limit stress—tangential (MPa)	*σ_c_*_,90,*T*,*y*_	20	10.2	4.0	39%
Modulus of elasticity—tangential (GPa)	*E_c_*_,90,*T*_	20	0.69	218	32%
Stress at 2mm deformation—tangential (MPa)	*σ_c_*_,90,*T*,2_	20	13.6	4.4	33%
***Static bending***					
Ultimate stress (MPa)	*σ_m_*	22	124.1	22.2	18%
Modulus of elasticity (GPa)	*E_m_*	22	22.27	4170	19%
***Shear parallel to grain***					
Ultimate stress (MPa)	*σ_v_*	44	16.2	3.0	19%
***Friction coefficient***					
0°	*μ_s_*_,0_	44	0.08	0.04	50%
45°	*μ_s_*_,45_	43	0.20	0.08	40%
90°	*μ_s_*_,90_	43	0.24	0.09	36%
